# Generation of a Recombinant scFv against Deoxycholic Acid and Its Conversion to a Quenchbody for One-Step Immunoassay

**DOI:** 10.3390/mps6050090

**Published:** 2023-09-30

**Authors:** Hiroshi Ueda, Hee-Jin Jeong

**Affiliations:** 1 Laboratory for Chemistry and Life Science, Institute of Innovative Research, Tokyo Institute of Technology, Yokohama 226-8503, Japan; ueda@res.titech.ac.jp; 2 Department of Biological and Chemical Engineering, Hongik University, Sejong 30016, Republic of Korea

**Keywords:** deoxycholic acid, single-chain variable fragment, recombinant antibody, Quenchbody, hapten, immunoassay

## Abstract

Development of a rapid detection method for deoxycholic acid (DCA) is crucial for its diagnosis in the early stages of inflammation and cancer. In this study, we expressed a soluble recombinant anti-DCA single-chain variable fragment (scFv) in *Escherichia coli*. To convert scFv into a Quenchbody (Q-body), we labeled scFv using commercially available maleimide-linked fluorophores. The TAMRA-C5-maleimide-conjugated Q-body showed the highest response within a few minutes of DCA addition, indicating its applicability as a wash-free immunoassay probe for onsite DCA detection.

## 1. Introduction

Deoxycholic acid (DCA) is a secondary bile acid that is a metabolic byproduct of intestinal bacteria [1]. Abnormally regulated DCA induces apoptosis of gastric epithelial cells, causes intestinal inflammation, and triggers gastric cancer [2,3,4]. Moreover, upregulated DCA in the intestinal tract promotes the excretion of chloride ions, increases intestinal permeability, and impedes mucosal healing, thereby causing irritable bowel disease [5]. Therefore, the rapid and accurate detection of DCA is essential for the diagnosis and monitoring of related diseases. Maekawa et al. developed a method for detecting DCA using electrospray ionization combined with tandem mass spectrometry using low-energy collision-induced dissociation [6]. Kakiyama et al. performed high performance liquid chromatography to determine DCA [7]. Although these methods provide high sensitivity, it is difficult to precisely detect the target molecules, particularly when the target molecule is mixed with other molecules with similar molecular weights to the target molecule. Moreover, chromatographic and mass spectrometric analyses have drawbacks, such as complexity, labor intensity, and requirements for large equipment and trained operators.

Antibodies have high specificity and selectivity against target molecules, and the time scale for antibody–antigen interactions is seconds to minutes. Therefore, antibody-based assays such as enzyme-linked immunosorbent assays (ELISA) enable the accurate and rapid quantification of antigens with high sensitivity. However, because the molecular weight of DCA is low (MW 393), its binding area to an antibody is narrow, which may cause difficulty in obtaining a high antibody–antigen affinity. Moreover, because such a small molecule, which is referred to as a hapten in immunochemical terms, is monovalent with a single epitope, it is generally difficult to use a sandwich ELISA that requires two epitopes to be bound by two antibodies at the same time [8,9]. Furthermore, haptens should be conjugated to a large carrier protein, such as bovine serum albumin (BSA), to be immobilized on a plate for performing direct or indirect ELISA, but such antigen-carrier protein conjugation is not appropriate for direct detection of an antigen itself. As an alternative method, a competitive ELISA can be used to detect a low-molecular-weight antigen with one epitope because one type of antibody is needed for the assay. However, competitive ELISA requires careful manipulation of the antibody and antigen concentrations to obtain a high signal-to-background ratio (S/B). Even though these ELISA-based approaches are more convenient than chromatography and mass spectrometry analyses, they require multiple washing and incubation steps, which makes the procedure time-consuming and labor-intensive, limiting their applicability. Therefore, the development of a novel immunoassay method for detecting DCA in a simple manner is required.

The versatility of fluorescence immunoassays has been widely expanded [10,11]. Among them, reagent-free fluorescence immunoassays offer the advantage of rapid and convenient measurements, which are comparable to traditional ELISA [12,13,14]. Reagent-free fluorescence immunoassays only require a few minutes for the full procedure composed of the single-step reaction of the fluorescent antibody and antigen because there is no need for additional steps, such as immobilization, washing, and secondary/tertiary antibody reactions. We have developed a powerful reagent-less fluorescence immunoassay probe named Quenchbody (Q-body) and have used it to detect various biomarkers [15,16]. We added a cysteine-containing peptide tag (Cys-tag) at the N-terminus of the antibody fragment domains, such as the antigen-binding fragment (Fab) and single-chain fragment variable (scFv), for site-specific labeling of the antibody [17,18]. Treatment of the terminal Cys-tag-linked antibody fragment with a mild reducing agent reduced the thiol group of the Cys-tag. Afterward, when a maleimide (mal)-conjugated fluorophore was added, the reduced thiol group of the Cys-tag was linked to the mal group via a maleimide-thiol reaction, resulting in a fluorophore-conjugated antibody. The fluorescence intensity of the Q-body increased because of the antigen-dependent removal of fluorophore quenching [19]. In the absence of an antigen, the fluorescence of rhodamine fluorophore was quenched by photo-induced electron transfer (PeT) from the indole side chain of tryptophan (Trp) or tyrosine residues in the antibody to the aromatic molecule of the dye [19,20,21,22]. However, when an antigen binds to the antibody, the conformation of the antibody variable region changes; thus, the dye near the antigen-binding site of the antibody is sterically distant from the antibody, leading to de-quenching of the dye [23]. In our previous studies regarding Q-bodies, mutants with the substitution of Trps in the variable domain of the antibody showed lower responses than those of the wildtype, indicating the efficiency of Trp residues in the quenching mechanism of Q-bodies [19,24]. It is worth noting that the entire assay for detecting the antigen occurs in a simple manner by adding a Q-body to the antigen-included sample, followed by measurement of the fluorescence intensity of the Q-body after a few minutes. This is comparable to conventional immunoassays, such as ELISA, which require several experimental steps, including immobilization, blocking, incubation, and washing. Based-on this Q-body-based simple and rapid immunoassay, we have detected various low molecular weight (MW) antigens, such as the BGP-C7 peptide (MW 894), phosphorylated vimentin PS71 and PS82 peptides (MW 1323 and 1372, respectively), methotrexate (MW 454), methamphetamine derivative (MW 166), aldosterone (MW 360), digoxin (MW 781), and 17β-estradiol (MW 272) [17,25,26,27,28,29,30]. Therefore, we assumed that the Q-body could be a potential reagent for DCA detection (MW 393).

In this study, we generated recombinant anti-DCA scFv and conjugated various fluorescent dyes with different properties and spacer lengths between maleimides and fluorophores. We selected the Q-body that showed the highest response in the presence of an antigen to demonstrate its potential use for the detection of DCA. Post-translational modifications (PTMs), such as glycosylation, are necessary to generate recombinant full-size IgGs that are typically used for in vivo diagnosis and treatment [31]. In contrast, recombinant antibody fragments such as scFv and Fab, which are widely used for in vitro diagnosis, do not require PTMs. Therefore, mammalian cells that enable PTMs of antibodies are used to express recombinant IgG, whereas *Escherichia coli*, whose handling is more cost-effective and has a lower contamination risk than mammalian cells, can be used to produce recombinant antibody fragments that do not require PTMs [32,33]. scFv is smaller and composed of a less complex structure than Fab. Moreover, the variable heavy chain (VH) and variable light chain (VL) domains of scFv were conjugated with a peptide linker, whereas the heavy (H) and light (L) chains of Fab were noncovalently bound to disulfide bonds. Therefore, Fab production presents multiple challenges, including low production yield with proper folding and equal expression of H and L chains [34]. In comparison, scFv provides a high yield with a suitable structure comprising the VH and VL domains in an equal ratio. Therefore, in this study, we produced an scFv-type antibody fragment against DCA using an *E. coli*-expression system.

## 2. Materials and Methods

### 2.1. Materials

KOD-plus-Neo and Ligation high were obtained from Toyobo (Osaka, Japan). Oligonucleotides were synthesized from Operon-Eurofins (Tokyo, Japan). Restriction enzymes and *E. coli* SHuffle T7 Express lysY were obtained from New England Biolabs Japan (Tokyo, Japan). Talon resin was obtained from Takara (Otsu, Japan). The 3 K ultra-filtration column was obtained from Pall (Ann Arbor, MI). TCEP gel was obtained from Thermo (Rockford, IL, USA). ATTO495-C2-mal, ATTO520-C2-mal, and ATTO655-Cx-mal (X is unknown) were obtained from ATTO-TEC (Siegen, Germany). R6G-C5-mal and TAMRA-C0-mal were obtained from Setareh Biotech Llc. (Eugene, OR, USA). TAMRA-C2-mal was obtained from Anaspec (Fremont, CA, USA). TAMRA-C5-mal was obtained from Biotium (Hayward, CA, USA). Cy3-C2-mal and Cy5-C2-mal were obtained from Kerafast (Boston, MA, USA). Rhodamine red-C2-mal was obtained from Life Technologies (Carlsbad, CA, USA). DCA was obtained from Sigma Japan (Tokyo, Japan). Other materials, unless otherwise indicated, were obtained from Wako (Osaka, Japan) or Sigma Japan.

### 2.2. Gene Cloning

The anti-DCA scFv sequence was based on a mouse monoclonal antibody against DCA [35,36]. pROX::anti-DCA scFv, which was a plasmid DNA where anti-DCA scFv-encoding DNA (kindly provided from Prof. Norihiro Kobayashi) was inserted to pROX vector [19], was amplified by PCR using primers, ProX_Age_GGSGG_back (5′-tctaatgagaccggtggcggttcaggtggc-3′) and T7t (3′-tagttattgctcagcg-5′), and KOD-Plus-Neo according to the manufacturer protocol (3-step version, Tm 43 °C). The amplified DNA was digested by AgeI and EagI and ligated using a Ligation high kit with AgeI- and EagI-digested pSQ::BGP [17], resulting in pSQ::anti-DCA scFv.

### 2.3. scFv Expression

SHuffle T7 Express lysY cells were transformed with pSQ::anti-DCA scFv and cultured at 30 °C for 16 h in LBA medium (LB medium containing 100 μg/mL ampicillin) with 1.5% agar. A single colony was selected and cultured overnight at 30 °C in 4 mL of LBA medium, then utilized to inoculate 100 mL of LBA medium. The cells were cultured at 37 °C until the OD_600_ reached 0.6; then, 0.4 mM isopropylthio-β-galactopyranoside (IPTG) was added and further incubated at 16 °C for 16 h. After centrifugation (3500 rpm, 20 min, 4 °C), the pellet was resuspended in 10 mL of lysis buffer (50 mM potassium phosphate, 300 mM sodium chloride, pH 7.4) and sonicated for 10 min with a pulse interval of 2 s. After centrifugation (3500 rpm, 4 °C, 20 min), the supernatant was incubated with 0.1 mL of Talon resin on a rotating wheel at room temperature (RT) for 1 h. The resin was washed thrice using 10 mL of wash buffer (50 mM phosphate, 300 mM sodium chloride, 5 mM imidazole, pH 7.4), followed by incubation with 5 mL of elution buffer (50 mM phosphate, 300 mM sodium chloride, 500 mM imidazole, pH 7.4) at RT for 1 h. The eluent was buffer exchanged to PBS using Nanosep Centrifugal-3 k ultrafiltration.

### 2.4. Q-Body Generation

A volume of immobilized TCEP disulfide reducing resin that was equivalent to the volume of 50 μg of purified protein was added to a microtube and centrifuged (100× *g*, 1 min, 4 °C). After removing the supernatant, 50 μg of purified protein was added and incubated for 1 h at RT on a rotating wheel. After centrifugation at 100× *g* for 1 min, the supernatant was recovered and reacted with 20 × mol of ATTO495-C2-mal, ATTO520-C2-mal, R6G-C5-mal, TAMRA-C0-mal, TAMRA-C2-mal, TAMRA-C5-mal, Cy3-C2-mal, Rho-C2-mal, Cy5-C2-mal, or ATTO655-Cx-mal in 2 μL of DMSO in the dark at RT for 2 h. Each mixture was incubated with 10 μL of His Mag Sepharose Ni beads, which is a highly crosslinked spherical agarose containing magnetite where Ni+ is linked to its surface (pore size of 37–100 μm, and 50 mg His-tagged protein/mL medium binding capacity) on a rotating wheel at RT for 30 min. The beads were washed thrice using 1 mL of His wash buffer (20 mM phosphate, 0.5 M NaCl, 60 mM imidazole, 0.1% polyoxyethylene(23)-lauryl ether, and pH 7.4) on a magnetic rack, which allowed capturing the magnetic beads within five seconds, resulting in the convenient and clear separation of the beads from the sample. After adding 500 μL of His elution buffer (20 mM phosphate, 0.5 M NaCl, 0.5 M imidazole, 0.1% polyoxyethylene(23)lauryl ether, and pH 7.4) and incubating at RT for 15 min, the eluent was collected on a magnetic rack and applied to Nanosep Centrifugal-3 k ultrafiltration. After equilibration twice using 500 μL of PBST by centrifuge (14,000× *g*, 20 min, 4 °C), the supernatant was concentrated to 200 μL. Then, 10 μL of each purified sample was mixed with 3 μL of SDS loading buffer (0.125 M Tris-HCl, 4% (*w*/*v*) SDS, 20% (*w*/*v*) glycerol, 0.01% (*w*/*v*) BPB, 100 mM DTT, and pH 6.8), boiled at 95 °C for 5 min, and loaded to SDS-PAGE gel. A fluorescence image was obtained using a transilluminator with excitation at 500 nm (Gelmiére, Wako, Osaka, Japan).

### 2.5. Fluorescence Measurement

First, 10 ng of Q-body in 250 μL of PBST was added in a 5 × 5 mm quartz cell (Starna Scientific, Hainault, UK), and a high concentration of DCA (3 μM) in PBST (2.5 μL of 300 μM DCA in PBST) was added and mixed with the Q-body solution. Three minutes after adding the antigen, the spectral measurement was performed at RT using the fluorescence spectrophotometer Model FP-8500 (JASCO, Tokyo, Japan). Both the excitation and emission slit widths were set to 5.0 nm. The excitation wavelength was 495, 520, 530, 546, 546, 546, 535, and 565 nm for the ATTO495-C2-mal-, ATTO520-C2-mal-, R6G-C5-mal-, TAMRA-C0-mal-, TAMRA-C2-mal-, TAMRA-C5-mal-, Cy3-C2-mal-, and Rho-C2-mal-labeled Q-body, respectively.

## 3. Results and Discussion

**Figure 1:**
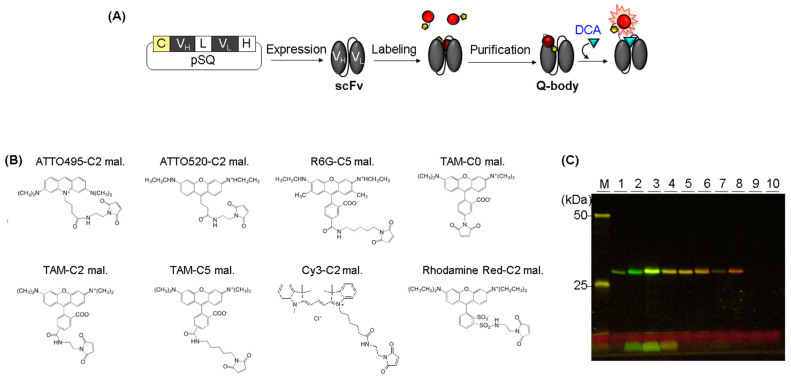
(**A**) Scheme for the construction of anti-DCA Q-body. (**B**) Chemical structure of the dyes for generating Q-body. (**C**) Fluorescence image of SDS-PAGE for Q-bodies. The estimated molecular weight of scFv, including tags was 28.2 kDa. M indicates a protein marker. 1, 2, 3, 4, 5, 6, 7, 8, 9, and 10 indicate the ATTO495-C2-mal-, ATTO520-C2-mal-, R6G-C5-mal-, TAMRA-C0-mal-, TAMRA-C2-mal-, TAMRA-C5-mal-, Cy3-C2-mal-, Rho-C2-mal-, Cy5-C2-mal-, and ATTO655-Cx-mal-labeled Q-bodies, respectively.

We expressed scFv in its soluble form in *E. coli* and purified it using Ni-affinity chromatography (Figure 1A). There are three Trps in the VH and two Trps in the VL, which are important residues for fluorophore quenching. We added a Cys-tag (SKQIEVNCSNET) at the N-terminus of scFv to introduce a Cys residue for site-specific conjugation of the thiol-reactive fluorescent dye to the terminus of the antibody. As dye modification should have not destroyed the three-dimensional structure of the protein, we added a labeling tag to the terminal region of scFv. In particular, dequenching of the dye in the Q-body is affected by the interaction between the antigen and antibody. Therefore, we introduced a Cys-tag at the N-terminus of scFv to conjugate a fluorescent dye near the antigen-binding site of the antibody. We labeled scFv with a range of fluorophores having different molecular and spectral properties to determine the best dye for generating anti-DCA Q-bodies. As Cy5 and ATT0655-conjugated Q-bodies were not detectable using a transilluminator in our lab, we used eight commercially available mal-conjugated dyes, whose emission wavelengths were in the detectable range of a transilluminator (Table 1, Figure 1B), including rhodamine derivatives, which are organic dyes substantially quenched by tryptophan or tyrosine residues [22]. As three commercial TAMRA-mal fluorophores with different spacer lengths between the fluorophore and maleimide (TAMRA-Cn-mal, n = 0, 2, 5, where Cn indicates the number of carbons between TAMRA and maleimide) were available, we compared their properties to address the effect of the distance between the fluorophore and Cys-tag. We labeled purified scFv with each dye using maleimide thiol reaction-based click chemistry [37]. SDS-PAGE analysis of the transilluminator revealed that fluorophore-labeled scFvs (Figure 1C). Unbound dyes were observed in ATTO520-C2-mal-, R6G-C5-mal-, and TAMRA-C0-mal-conjugated Q-body samples that remained after purification, whereas the unbound dye in the other five Q-body samples was eliminated even after the labeling and purification of eight Q-bodies were performed simultaneously. Although the exact reason for the observed differences in the properties of the ATTO520-C2-mal-, R6G-C5-mal-, and TAMRA-C0-mal-conjugated Q-bodies is difficult to explain, the charge and hydrophobicity of the dye may affect nonspecific binding [38]. Hydrophobicity is represented by the distribution coefficient LogD, which is the propensity of a dye to prefer polar or nonpolar solvents [38,39]. Because R6G is a hydrophobic dye with a positive LogD value and easily forms aggregates [40], the excess R6G was nonspecifically adsorbed onto the purification resin even after washing and then detached during the elution process. The unbound TAMRA-C0-mal was not fully eliminated, whereas those of TAMRA-C2-mal and TAMRA-C5-mal were clearly eliminated. We assumed that the shorter linker length between the dye and maleimide might reduce the aggregation of the dye before reacting with the antibody. Compared with TAMRA and R6G, ATTO520 is less hydrophobic. However, the ATTO520-C2-mal-conjugated Q-body contained more free dye than the TAMRA-C2-mal, which had the same spacer length. ATTO520 is a positively charged fluorophore, which normally causes nonspecific staining, but there is another statement that a correlation between charge and nonspecific binding is low [38,39,41]. Although clear evidence of this nonspecific binding could not be presented in the current study, we plan to perform a followup study to further clarify the dye-relevant labeling and purification efficiency by increasing the variations of antibodies and dyes, including not only these commercially available maleimide-conjugated fluorophores but also synthetic organic dyes to enlarge the pools of antibodies and dyes.

**Figure 2:**
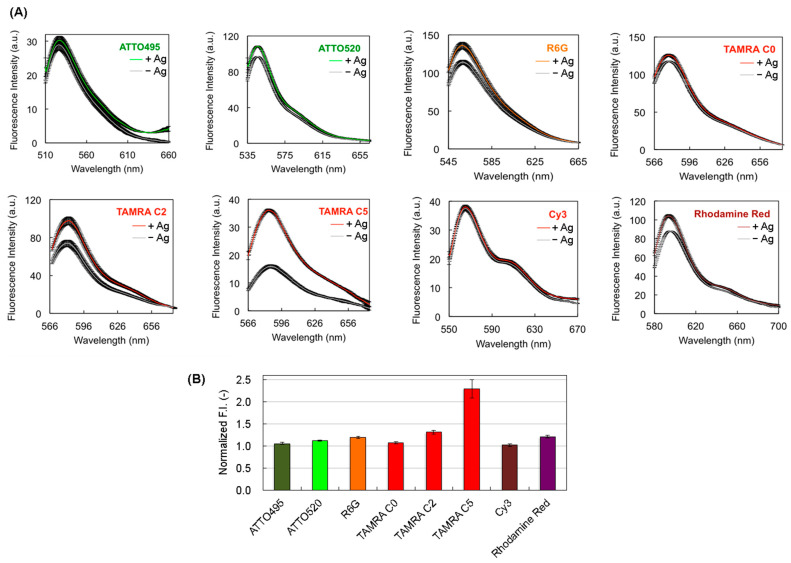
(**A**) Fluorescence spectra of Q-bodies in the presence or absence of 3 μM of DCA. The black shaded area represents ±1 standard deviation (SD) (n = 3); (**B**) Normalized fluorescence intensities of Q-bodies. Error bars represent ±1 SD (n = 3).

Each Q-body was mixed with an antigen, and its fluorescence intensity was measured. In the presence of DCA, the fluorescence intensities of Q-bodies labeled with ATTO495-C2-mal, ATTO520-C2-mal, R6G-C5-mal, TAMRA-C0-mal, TAMRA-C2-mal, TAMRA-C5-mal, Cy3-C2-mal, and Rho-C2-mal increased 1.05 ± 0.03-, 1.12 ± 0.01-, 1.19 ± 0.03-, 1.07 ± 0.02-, 1.31 ± 0.04-, 2.29 ± 0.21-, 1.02 ± 0.03-, and 1.20 ± 0.03-fold, respectively (Figure 2). The signals varied between dyes, and the highest S/B was observed for the TAMRA-C5-mal-conjugated Q-body. The order of the responses differed from other results for Q-bodies produced using various dyes (Table 1). This indicates that the fluorescence response is dependent on the fluorophore of each antibody. Although the explanation for this response is currently unknown, we estimated that the different distances between the dye and Trp residues of each antibody and the different numbers of Trp residues near the dye might cause a variant fluorescence response. Thus, it is important to test the best fluorophore from the dye library, including characteristically unknown dyes, to select a Q-body with a high response. Although the reason for this different response to the dye remains unclear, we herein show that TAMRA-C5 is the best dye for generating an anti-DCA Q-body. The hydrophobicity of the dye can be an important factor for labeling the antibody, followed by the purification of the excess unbound dyes. When the excess dye is not clearly removed but remains in the Q-body solution, the background signal of the Q-body in the absence of antigen becomes high, causing a low S/B of the Q-body response. Therefore, it is possible that the response of ATTO520-C2-mal, R6G-C5-mal, and TAMRA-C0-mal-conjugated Q-bodies could be improved if clearer purification, such as subsequent second purification using gel chromatography, is performed. However, the larger the number of purifications, the lower the yield because the purification step causes a loss of the sample. In this study, we focused the research point on the expression of recombinant anti-DCA scFv and determined the best dye among commercially available maleimide-conjugated fluorophores for creating a Q-body. Therefore, we investigated whether the TAMRA-C5-mal-conjugated anti-DCA Q-body could be used as a probe to detect DCA. There were no washing or blocking steps necessary, but mixing the Q-body with the antigen was sufficient to measure the fluorescence response in a few minutes, indicating the usefulness of this Q-body for a one-pot reagent-less immunoassay.

## 4. Conclusions

In this study, we successfully generated a recombinant scFv against DCA, whose abnormal regulation causes cancer and inflammation. We produced an *E. coli*-based scFv within four days, promoting the extension of its application to other DCA detection-related areas. We synthesized Q-bodies against DCA using Cys-tagging-based site-specific post-labeling with a recombinant anti-DCA scFv. We labeled the Q-body with several fluorophores with different spacer lengths between the maleimide and the dye and examined their properties. The variation in the fluorescence response was dependent on the fluorophore, which reflects the differences in their de-quenching behaviors. The fluorescence response of the TAMRA-C5-mal-labeled Q-body was 2.3-fold in the presence of DCA, which was the highest response among the fluorophores examined. It is worth noting that the Q-body response occurred within a few minutes after adding DCA, and there was no additional step; only mixing of the Q-body and antigen was needed. Moreover, a small antigen was detected using this Q-body-based system without any carrier protein conjugation to the antigen. The aim of this study was to generate anti-DCA scFv using *E. coli* and to select a fluorescence dye that is appropriate for converting scFv to Q-body. Further experiments will be conducted to reveal the detailed properties of this Q-body, including its sensitivity and selectivity, in both in vitro and in vivo systems. Its potential applications are extensive for the executive detection and diagnosis of DCA-related diseases.

## Figures and Tables

**Table 1:** Summary of the dye characteristics. The logarithm of the distribution coefficient (LogD) was calculated from the structure of dye using Marvin Sketch software (Chemaxon, Version 23.10). ND means Not Determined.

Dye	Ex_max_ (nm)	Em_max_ (nm)	LogD at pH 7.4	Net Charge at pH 7.4	Fluorescence Intensity			
					Anti-DCA scFv (This Study)	Anti-BGP ^1^ scFv [17]	Anti-ICP ^2^ Fab [42]	Anti-Lysozyme VH [43]
ATTO495-C2-mal.	495	525	−2.63	−0.81	1.05	1.1	≈0.9	ND
ATTO520-C2-mal.	520	546	−1.31	−0.98	1.12	2.7	≈1.0	≈3.3
R6G-C5-mal.	530	558	+5.59	−1.70	1.19	5.0	2.6	≈4.5
TAMRA-C0-mal.	546	578	+4.59	−1.04	1.07	2.9	≈1.5	≈1.6
TAMRA-C2-mal.	546	583	+3.81	−1.31	1.31	2.0	≈1.9	≈1.4
TAMRA-C5-mal.	546	588	+4.83	−1.12	2.29	4.0	≈1.1	≈3.2
Cy3-C2-mal.	535	566	+2.76	−0.86	1.02	ND	ND	ND
Rho. Red-C2-mal.	565	592	+1.87	−1.34	1.20	1.7	ND	ND

^1^ bone g-carboxyglutamic acid-protein. ^2^ imidacloprid.

## Data Availability

The data presented in this study are available on request from the corresponding author.
